# Hidradenitis Suppurativa in the SARS-CoV-2 Pandemic: Investigation of Trigger Factors in a Single Center

**DOI:** 10.3390/jcm13144074

**Published:** 2024-07-12

**Authors:** Lisa Scholl, Nessr Abu Rached, Eggert Stockfleth, Philipp Cramer, Lennart Ocker, René Stranzenbach, Simone Garcovich, Schapoor Hessam, Falk G. Bechara

**Affiliations:** 1 International Centre for Hidradenitis Suppurativa/Acne inversa (ICH), Department of Dermatology, Venereology and Allergology, Ruhr-University Bochum, 44791 Bochum, Germany; eggert.stockfleth@kklbo.de (E.S.); falk.bechara@klinikum-bochum.de (F.G.B.); 2 Skin Cancer Center, Department of Dermatology, Venereology and Allergology, Ruhr-University Bochum, 44791 Bochum, Germany; 3 Dermatology Outpatient Service Cersid, 00100 Rome, Italy

**Keywords:** hidradenitis suppurativa, HS, acne inversa, trigger factors, symptoms, pandemic, COVID-19, SARS-CoV-2, diet change

## Abstract

**Background:** Hidradenitis suppurativa (HS) is a debilitating, chronic inflammatory disease associated with multiple triggers. As the world struggles with the global COVID-19 pandemic, it is important to review the trigger factors for chronically ill HS patients during the COVID-19 pandemic. This work investigates the self-described trigger factors of HS patients that emerged during the COVID-19 outbreak. **Methods**: We anonymously surveyed 110 HS patients during the SARS-CoV-2 pandemic using a 25-question questionnaire that included trigger factors for deterioration. Demographic, personal, and HS-specific information was also collected to identify potential trigger factors for HS exacerbation. All HS patients were asked if their HS had worsened compared to the time before the pandemic. **Results:** Compared to before the pandemic, 20% of HS patients (*n* = 22) reported a worsening of HS. Patients with an HS exacerbation were significantly more likely to avoid contact with a doctor than those without an exacerbation (45.5% vs. 18.2%; *p* = 0.007). HS involvement, severity, exercise activity, and BMI had no association with worsening HS (*p* > 0.05). Interestingly, dietary changes and increased consumption of sweets and treats were associated with worsening HS (*p* = 0.011 and *p* = 0.013). Specifically, eating more sweets and treats was associated with a 6-fold increased risk of worsening HS. The results suggest that diet has an important influence on HS relapses. Further investigation is needed to determine whether diet is a triggering factor independent of the SARS-CoV-2 pandemic. In addition, gluteal HS involvement was associated with a more than 4.3-fold risk of HS exacerbation. **Conclusions:** In the management of HS patients, it is important to consider that gluteal involvement and the consumption of sweets are more often associated with deterioration.

## 1. Introduction

Hidradenitis suppurativa (HS), a painful and often disfiguring skin condition, has garnered increased attention from the medical community in recent years. This chronic skin disease is characterized by inflamed abscesses, fistulas, and nodules in areas of the skin containing hair follicles, imposing a significant burden on those affected [1,2]. HS inflammation is also expressed in the blood. For example, disease severity correlates with haptoglobin levels in the blood [3]. A genetic and epigenetic cause is suspected in the pathogenesis of HS [4,5]. Wet shaving, an unhealthy diet, smoking, and obesity are thought to be triggering factors [6,7]. Recently, the role of physical inactivity and poor diet was discussed in patients with HS [8]. Nutrition plays a key role in modulating inflammatory processes and can therefore have a significant impact on chronic inflammatory diseases such as HS [9,10,11,12]. During the pandemic, lockdowns, restrictions on food availability, and dietary changes led to significant nutritional changes for many people [13,14]. These changes in diet may increase the severity and frequency of HS episodes.

In addition to the known factors that could trigger or exacerbate HS, the COVID-19 pandemic introduced a new dimension. The repercussions of the pandemic, including social isolation, stress, and lifestyle changes, emerged as potential trigger factors for HS episodes, placing this condition under the spotlight of research and medical care. In this article, we will take a closer look at the relationship between hidradenitis suppurativa and the severe acute respiratory syndrome coronavirus 2 (SARS-CoV-2) pandemic, specifically focusing on the potential trigger factors that influenced the symptoms of this condition. The aim of this study was to investigate how the lifestyle changes during the pandemic had an impact on the worsening of HS. Additionally, reduced physical activity during the lockdown period led to an increase in physical inactivity and obesity, both of which are known risk factors for HS worsening [15]. Apart from the direct impact on diet and physical activity, the COVID-19 pandemic also had an indirect impact on the health of HS patients. Limited access to medical and dermatological care during the lockdowns made disease management much more difficult.

To our knowledge, the trigger factors during the SARS-CoV-2 pandemic have never been analyzed. These findings should help to develop future treatment strategies and preventive measures that can improve people’s quality of life and reduce the burden of disease in HS.

## 2. Materials and Methods

### 2.1. Study Design, Patients, and Data Collection

During the first phase of a nationwide lockdown, which started on 25 March 2020, HS patients in the International Centre for Hidradenitis suppurativa/Acne inversa (ICH) at the Department of Dermatology Bochum received an anonymous questionnaire. The anonymous questionnaires were collected in May 2020. The survey data were analyzed retrospectively. A total of 110 HS patients were recruited in May 2020. Recruitment was consecutive and randomized, regardless of disease stage or treatment plan, with no predefined inclusion criteria, defined as non-probabilistic convenience sampling. Trained staff interviewed patients who attended the department for routine visits. As only anonymized data were used and no pseudo-anonymization was applied, it was not possible to identify the patients through the questionnaire.

Demographic and clinical characteristics of the patients were recorded on the survey form. Clinical manifestations were classified into three groups using the Hurley stage. Inflammatory episodes were defined as new-onset or worsening of pre-existing skin lesions with physical signs of heat, redness, swelling, and pain [16].

The validated German version of the DLQI questionnaire was used to assess the quality of life [17].

### 2.2. Study Questionnaire

A questionnaire was developed by the group of authors of this article and consisted of 25 questions. To manage patient care during the pandemic and reduce face-to-face contact time, the questionnaire was developed. The questionnaire items were structured to capture patient demographics and the impact of COVID-19 closure measures on disease outlook, access to and compliance with clinical care before and during closure, as well as the impact on well-being, medical support, and treatment compliance. The impact on disease-specific quality of life was assessed using the Dermatology Quality of Life Index (DLQI) [17]. This study was conducted in accordance with the tenets of the Declaration of Helsinki [18].

### 2.3. Statistical Analysis

Normal distribution was tested by the Shapiro–Wilk test and a Q-Q plot for all variables. To determine the interquartile range (IQR), the difference between the 3rd quartile and the 1st quartile was calculated. Parametric and non-parametric tests (including the Chi^2^ test and Mann–Whitney U test) were used to determine differences between groups. To examine the influence of multiple variables on HS exacerbation, we performed logistic regression with all variables (*p* < 0.01) as well as all variables that may have an influence on HS exacerbation. Statistical analyses were performed using IBM SPSS Statistics (version 29.0.0.0, New York, NY, USA, 2022). A *p*-value < 0.05 was considered statistically significant.

## 3. Results

### 3.1. Personal and Clinical Characteristics of HS Patients

Table 1 provides a comprehensive overview of the demographic and clinical characteristics of a cohort of 110 people with hidradenitis suppurativa (HS) interviewed during COVID-19. Fifty-nine patients (53.6%) were female and 51 (46.4%) were male, with a median age of 41 years (ICR, 31–51.8). Forty-one (37.3%) of the patients were current smokers. The median body mass index (BMI) was 30.1 kg/m^2^ (ICR 26.9–35.4). In addition, the median disease duration was 14 years (ICR 8–22.8). Nine patients were Hurley stage I (8.2%), 49 were Hurley stage II (44.6%), and 52 were Hurley stage III (47.3%). Furthermore, the table explores the patients’ subjective assessment of their HS condition, with 20% reporting deterioration and 80% indicating no deterioration.

### 3.2. Impact of HS-Specific Characteristics on HS Deterioration

Subsequently, we performed a comparative analysis of specific characteristics associated with the exacerbation of hidradenitis suppurativa (HS) symptoms (Table 2). As expected, during the pandemic, patients with HS exacerbation reported significantly more pain than patients without HS exacerbation (50% vs. 5.7%; *p* < 0.001). Patients with an HS exacerbation were significantly more likely to avoid contact with a doctor than those without an exacerbation (45.5% vs. 18.2%; *p* = 0.007). BMI, age, current smoker, disease duration, and Hurley III were not significantly associated with HS exacerbation during the pandemic (*p* > 0.05). Involvement of specific anatomical regions also had no association with worsening of HS symptoms (*p* > 0.05).

### 3.3. Impact of Lifestyle Changes on HS Exacerbation

In addition, to investigate the influence of lifestyle factors, we compared lifestyle changes in patients with HS during the pandemic, distinguishing between those with worsening HS symptoms and those without worsening symptoms (Table 3). Significantly, 63.6% of HS patients with worsening symptoms reported making dietary changes, compared to 34.1% of those without worsening symptoms (*p* = 0.011). A total of 36.4% of HS patients with worsening symptoms reported increased consumption of sweets and treats, which was statistically significant (*p* = 0.013). A higher percentage of patients with worsening HS (18.2%) reported an increase in fast food consumption, though the difference was not statistically significant (*p* = 0.1). There was no association between meat and vegetable consumption and HS exacerbation (*p* > 0.05). Both groups experienced reduced physical activity, with 40.9% of those with worsening HS and 33% of those without worsening HS reporting less sport and movement (*p* = 0.48). The median daily sport exercise during the pandemic was similar in both groups (*p* = 0.82). Median weight gain was negligible in both groups, with no significant variation (*p* = 0.63). A small percentage of patients in both groups reported changes in their relationship with their partner (9.1% vs. 6.8%), with no statistical significance (*p* = 0.7).

### 3.4. Comparison of Medication and Ongoing Therapies from HS Patients

We conducted a comparison of medication and ongoing therapies in our cohort (Table 4). In total, 40.9% of both HS groups with worsening symptoms and patients without worsening symptoms reported not receiving treatment during the pandemic (*p* > 0.05). Other therapies such as oral zinc gluconate, antibiotics, and biologics also showed no association with worsening HS (*p* > 0.05).

### 3.5. Logistic Regression Analysis for the Detection of Independent Trigger Factors

**Figure 1:**
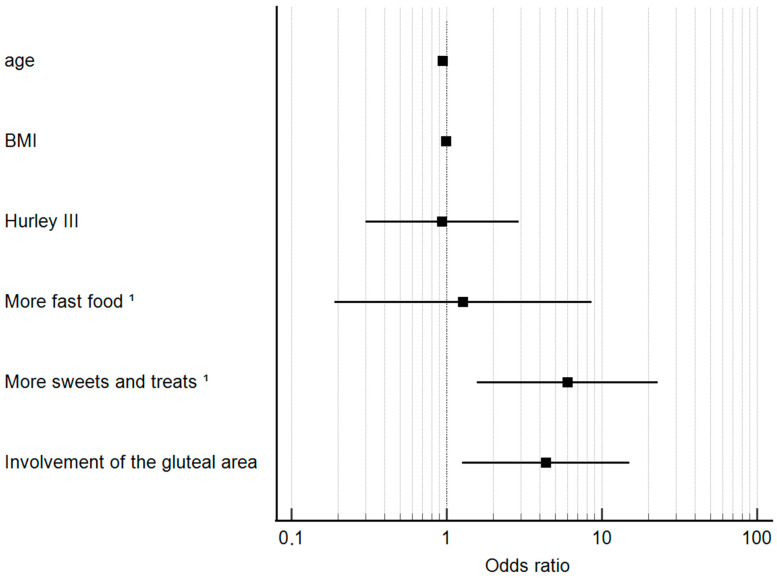
Odds ratio plot showing trigger factors that had an impact on worsening HS during the COVID-19 pandemic. In particular, more sweets and treats and gluteal involvement of HS were significantly associated with worsening HS. ^1^ than before the pandemic.

All variables with a *p*-value < 0.1 and all factors that could influence HS worsening were included in the logistic regression analysis (Table 5 and Figure 1). Lower age was associated with a reduced likelihood of HS exacerbation during the COVID pandemic (OR 0.945; 95% Confidence Interval [CI] 0.9–0.991; *p* = 0.021). Interestingly, consuming more sweets and treats during the pandemic was associated with 6-fold increased odds of HS exacerbation (OR 6.01; 95% CI 1.57–23; *p* = 0.009). There was also a significant association between buttock involvement and HS worsening (OR 4.36; 95% CI 1.26–15.1; *p* = 0.02). In the regression model, the variables BMI, Hurley III, and an increased consumption of fast food were not associated with HS worsening (*p* > 0.05).

## 4. Discussion

On 25 March 2020, the German government enacted emergency laws and restrictions in hospitals and outpatient clinics due to the global spread of SARS-CoV-2 [19]. These restrictions had a major impact on the lifestyle of patients, especially patients with HS. The COVID-19 pandemic restrictions changed people’s lifestyles [20,21]. In addition, people moved less because of the restrictions, so this could also have an impact on HS [22]. In this retrospective study, we aimed to investigate the impact of the COVID-19 pandemic on trigger factors in a typical cohort of HS patients managed in a specialized HS center. To our knowledge, the triggering factors of HS during the COVID-19 pandemic have not been studied.

Miller et al. reported that HS is associated with high fat, high visceral fat, and low muscle [23]. Symptoms and severity of HS are influenced by BMI and adipose tissue [24,25]. However, no prospective studies have investigated sport and exercise in HS. This gap should be filled by new studies in the future. Our studies showed no association between weight gain and HS exacerbation. However, our patients did not gain much weight during the COVID-19 pandemic (median 0 kg; range −8 to +25), so conclusions in this regard are limited. The BMI values were also not significantly different between the HS worsening and non-worsening groups, so the influence of BMI on HS worsening during the pandemic is limited. A longitudinal study should investigate the effect of BMI on HS patients. On the other hand, there are reports of a beneficial effect of bariatric surgery and weight loss on HS symptoms [6,26].

Less sport and exercise were not a significant variable for HS deterioration in our analysis. As a result of the reduction in movement, people were more sedentary. Interestingly, gluteal HS involvement was significantly associated with a 4.36-fold increased risk of HS worsening (OR 4.36; 95% CI 1.26–15.1; *p* = 0.02). Prolonged sitting causes HS patients to experience more mechanical stress in the gluteal region. This proves that friction, mechanical forces, and pressure are important trigger factors for HS [27,28]. In addition, prolonged sitting could inhibit the flow of drainage fistulas in the gluteal region, which could lead to more severe relapses.

Compared to patients without dietary changes, patients with dietary changes were significantly more likely to have HS exacerbations (63.9% vs. 34.1%; *p* = 0.011). Naik et al. [29] reported that sweets, bread, pasta, rice, dairy products, and high-fat foods are among common trigger factors of HS. In our analysis, we were able to report that consuming more sweets and treats was an important triggering factor in the COVID-19 pandemic. There was a 6-fold increased risk of worsening HS if the patient ate more sweets and treats. One possible explanation is that increased sugar consumption may increase insulin resistance. This could lead to an overall increase in inflammation. The link between these two variables remains speculative. Furthermore, nutrition stimulates insulin and IGF-1 (insulin-like growth factor 1), which influence signaling pathways involved in the pathogenesis of HS [12]. A possible link between HS and the consumption of ultra-processed foods is discussed in the literature [11]. In a case-control study in the Netherlands, poor diet quality and lower physical activity were significantly more frequently associated with HS in the general population [8]. We suggest that in the future, it will be important to study the diet of HS patients to identify more accurate dietary components. One limitation is that we only included a few foods to keep the sheet manageable. In our analysis, we did not find a positive effect of dietary changes in HS patients.

A major factor for HS deterioration is represented by HS severity. During the pandemic, HS severity as measured by Hurley staging was not a significant factor. However, a limitation of the Hurley system is that it does not adequately represent inflammatory activity, so, for example, the IHS4 or SAHS score would be more appropriate [30,31]. Another option for measuring the severity of HS in the future may be ultrasound-based classification [32]. During the pandemic, HS exacerbations were not affected by different ongoing therapies in HS patients.

Some limitations of this study are its retrospective design, the use of remote data capture, and the anonymized structure of the survey, which restricted the collection of further information during the pandemic period. Despite the limitations of the study design, this study provides new findings that are important for further HS research.

## 5. Conclusions

Our analyses showed that diet was an important trigger of HS during the COVID-19 pandemic. Specifically, eating more sweets and treats was associated with a 6-fold increased risk of worsening HS. In addition, gluteal HS involvement was associated with a more than 4.3-fold risk of HS exacerbation. In the treatment management of HS, it is important to consider that gluteal involvement and the consumption of sweets are more often associated with HS deterioration. In the future, a study on the role of dietary patterns should be conducted to identify potential trigger factors of HS disease.

## Figures and Tables

**Table 1:** Personal and clinical characteristics of HS patients (*n* = 110).

Parameter		Value (s)
sex, *n* (%)	female	59 (53.6)
	male	51 (43.4)
age, median (ICR), y		41 (31–51.8)
disease duration, median (IQR), y		14 (8–22.8)
BMI, median (ICR), kg/m^2^		30.1 (26.9–35.4)
smoker, *n* (%)	current smoker	41 (37.2)
	not specified	9 (8.2)
	non-smoker	60 (54.6)
subjective deterioration of HS, *n* (%)	Yes	22 (20)
	no	88 (80)
Hurley classification, *n* (%)	Hurley I	9 (8.2)
	Hurley II	49 (44.6)
	Hurley III	51 (47.2)

*n*, absolute number of patients; y, years; IQR, interquartile range; BMI, body mass index; HS, hidradenitis suppurativa.

**Table 2:** Comparison of HS-specific characteristics with worsening of HS symptoms.

Parameter	HS Patients with Worsening HS (*n* = 22)	HS Patients without Worsening HS (*n* = 88)	*p* Value
Male vs. female, *n* (%)	12 (23.5) vs. 10 (17)	39 (76.5) vs. 49 (83.1)	0.39
BMI, median (ICR), kg/m^2^	31.6 (27.7–35.3)	30.1 (26.5–35.3)	0.5
age, median (ICR), y	35 (27.3–47.8)	42.5 (32.5–52.3)	0.08
current smoker, *n* (%)	7 (31.8)	34 (38.6)	0.18
Disease duration, median (ICR), y	12 (7–23)	14.5 (8–22.3)	0.62
Hurley III, *n* (%)	11 (50)	41 (46.6)	0.8
More pain than before the pandemic, *n* (%)	11 (50)	5 (5.7)	<0.001 *
Avoidance of visits to the doctor, *n* (%)	10 (45.5)	16 (18.2)	0.007 *
Involvement of the axillary region, *n* (%)	8 (22.2)	14 (18.9)	0.7
Involvement of the inguinal and thigh area, *n* (%)	18 (81.2)	64 (72.7)	0.38
Involvement of the gluteal area, *n* (%)	11 (50)	25 (28.4)	0.05
Involvement of the perianal or perineal area, *n* (%)	9 (40.9)	32 (36.3)	0.69
Involvement of the genital area, *n* (%)	7 (31.8)	38 (43.1)	0.33
Involvement of the Mons pubis, *n* (%)	4 (18.2)	26 (29.6)	0.28
Involvement of atypical localizations, *n* (%)	0 (0)	13 (14.8)	0.06

*n*, absolute number of patients; y, years; IQR, interquartile range; BMI, body mass index; HS, hidradenitis suppurativa; * significant result.

**Table 3:** Comparison of lifestyle changes in HS patients (*n* = 110) with and without worsening HS during the pandemic.

Parameter	HS Patients with Worsening HS (*n* = 22)	HS Patients without Worsening HS (*n* = 88)	*p* Value
Diet change, *n* (%)	14 (63.6)	30 (34.1)	0.011 *
More fast food ^1^, *n* (%)	4 (18.2)	6 (6.8)	0.1
More meat ^1^, *n* (%)	1 (4.6)	1 (1.1)	0.28
More vegetables ^1^, *n* (%)	5 (22.7)	13 (14.8)	0.37
More sweets and treats ^1^, *n* (%)	8 (36.4)	12 (13.6)	0.013 *
Less sport and movement ^1^, *n* (%)	9 (40.9)	29 (33)	0.48
Sport activity per day ^2^, median (ICR), h	2 (1–8.5)	2.25 (1–8)	0.82
Weight gain ^1^, median (range), kg	0 (−8 to +25)	0 (−20 to +25)	0.63
Relationship with the partner has changed ^1^, *n* (%)	2 (9.1)	6 (6.8)	0.7

*n*, absolute number of patients; IQR, interquartile range. ^1^ than before the pandemic; ^2^ during the pandemic; *, significant result.

**Table 4:** Comparison of medication and ongoing therapies from HS patients (*n* = 110) with and without worsening HS during the pandemic.

Parameter	HS Patients with Worsening HS (*n* = 22)	HS Patients without Worsening HS (*n* = 88)	*p* Value
No therapy ^1^, *n* (%)	9 (40.9)	36 (40.9)	>0.9
Oral zinc gluconate with triclosan local therapy, *n* (%)	2 (9.1)	6 (6.8)	0.7
Antibiotics with clindamycin and rifampicin, *n* (%)	0 (0)	4 (4.6)	0.31
Antibiotic therapy, *n* (%)	0 (0)	5 (5.7)	0.25
Ongoing therapy with biologic, *n* (%)	11 (50)	41 (46.6)	0.8

*n*, absolute number of patients. ^1^ except local therapies.

**Table 5:** Logistic regression analysis for the detection of independent trigger factors; inclusion of all variables with a *p*-value < 0.1 and all factors that could influence HS worsening.

Parameters	Odds Ratio (OR)	95% Confidence Interval (CI)	*p* Value
age	0.945	0.9–0.991	0.021 *
BMI	0.99	0.92–1.07	0.8
Hurley III	0.93	0.3–2.93	0.9
More fast food ^1^	1.27	0.19–8.64	0.8
More sweets and treats ^1^	6.01	1.57–23	0.009 *
Involvement of the gluteal area	4.36	1.26–15.1	0.02 *

BMI, body mass index. ^1^ than before the pandemic. *, significant result.

## Data Availability

The data presented in this study are available upon request from the corresponding author. The data are not publicly available due to privacy restrictions.
